# Barriers and Facilitators to Patient Education Among Nurses in Multicultural Hospital Settings: A Cross-Sectional Study

**DOI:** 10.3390/nursrep15100371

**Published:** 2025-10-17

**Authors:** Hawazen Omar Rawas, Jennifer de Beer, Siti Awa Abu Bakar, Sarah Almutairi, Nehal Jaafari, Hawazen Hazzazi, Asma Alzahrani, Raghad Alghumuy, Najwa Hadadi, Sarah Alfahimi, Samar Alharbi, Elham Yahya Alzubaidi, Ahmad Rajeh Saifan, Nabeel Al-Yateem

**Affiliations:** 1 Nursing Department, College of Medicine and Health Sciences, Arabian Gulf University, Manama P.O. Box 26671, Bahrain; hawazenor@agu.edu.bh; 2 King Faisal Specialist Hospital & Research Center, Jeddah 12713, Saudi Arabia; debeerj@kfshrc.edu.sa; 3 Department of Pediatric Nursing, College of Nursing, King Saud bin Abdulaziz University for Health Sciences, Jeddah 21423, Saudi Arabia; bakars@ksau-hs.edu.sa (S.A.A.B.); 421220587@ksau-hs.edu.sa (S.A.); 421220641@ksau-hs.edu.sa (H.H.); 421220505@ksau-hs.edu.sa (A.A.); 421220551@ksau-hs.edu.sa (R.A.); 421220628@ksau-hs.edu.sa (N.H.); 421220586@ksau-hs.edu.sa (S.A.); alharbi0583@ksau-hs.edu.sa (S.A.); 421220511@ksau-hs.edu.sa (E.Y.A.); 4 Faculty of Nursing, Yarmouk University, Irbid 21163, Jordan; 5 Department of Nursing, College of Health Sciences, University of Sharjah, Sharjah P.O. Box 27272, United Arab Emirates

**Keywords:** cultural competency, cross-sectional studies, nursing, patient education, quality of health care, nursing workforce, healthcare barriers, facilitators, cultural competence, Saudi Arabia

## Abstract

**Background:** Patient education (PE) is an essential component of quality healthcare and chronic disease management. However, effective implementation often faces patient-, nurse-, and organization-related barriers. This is particularly relevant in multicultural healthcare settings such as Saudi Arabia, where a highly diverse nursing workforce may influence PE practices. **Aim:** To examine the barriers and facilitators influencing patient education practices among nurses working in multiple hospitals in Saudi Arabia. **Methods:** A descriptive cross-sectional study was conducted among 289 registered nurses recruited through convenience sampling from various hospitals in Saudi Arabia. Data were collected using a validated self-administered questionnaire consisting of demographic items and structured scales assessing PE barriers and facilitators. Descriptive statistics were used to analyze the data. **Results:** Language differences (64.3%) and cultural barriers (59.2%) were the most commonly reported patient-related obstacles. Among nurse-related barriers, staff shortages (72.4%), heavy workload (72.0%), and time constraints (59.9%) were prominent. Organizational barriers included limited educational resources (39.4%) and unsupportive environments (35.6%). Key facilitators identified by nurses included availability of policies and procedures (63.6%), provision of PE training (63.7%), and integration of PE into clinical workflow and nurse appraisals. **Conclusions:** Despite strong professional support for PE, multiple barriers hinder its implementation in Saudi hospitals. Addressing these challenges requires institutional strategies such as workforce reinforcement, policy integration, and resource allocation. Future efforts should also include integrating patient perspectives, developing culturally tailored education resources, and evaluating the impact of targeted interventions to strengthen PE delivery in diverse hospital settings.

## 1. Introduction

Effective patient education (PE) is a cornerstone of quality healthcare, significantly influencing health outcomes, treatment adherence, and patient empowerment. It involves the purposeful delivery of health-related information to patients and their families to promote behavior change, support self-care, and encourage optimal health practices [1]. To be effective, education must be tailored to the individual, delivered in patient-friendly language, and designed to enable patients to participate actively in their care, thereby reducing complications and avoidable hospital readmissions [1,2].

Extensive research underscores the value of PE in chronic disease management. Evidence shows that standardized, structured education programs improve knowledge, adherence, self-efficacy, and psychological outcomes across a wide range of chronic conditions—including cardiovascular disease, diabetes, cancer, asthma, and communicable diseases such as tuberculosis and hepatitis C [3,4]. Patient-centered and group-based approaches have also proven effective in enhancing emotional well-being, self-monitoring, communication with healthcare providers, and overall quality of life [5,6]. However, nonadherence remains a persistent challenge, driven by multifactorial elements such as patient beliefs, treatment complexity, illness perceptions, and healthcare relationships [7,8] Recent advances in mobile and digital health technologies offer new tools to support PE and improve adherence. When integrated with personalized education strategies and patient trust, digital systems have been shown to increase disease-specific knowledge, self-management capabilities, and behavioral compliance—particularly in remote and resource-limited settings [9,10]. These innovations promise scalable and cost-effective solutions, but successful implementation depends on healthcare providers’ ability to deliver education that is tailored, culturally competent, and contextually grounded [11,12].

Despite its benefits, the implementation of PE faces several well-documented barriers. These include patient-related issues (e.g., language and cultural gaps, low motivation, health beliefs), nurse-related factors (e.g., time constraints, limited training, workload), and organizational challenges (e.g., lack of resources, unsupportive environments) [13,14,15]. Facilitators, such as integrating PE into nursing appraisals, allocating teaching time, and providing accessible materials and technology, can enhance delivery and mitigate these obstacles [16,17].

The Saudi Arabian healthcare system presents a particularly complex environment for PE implementation [18,19]. One of its defining features is the highly diversified healthcare workforce, with nurses and healthcare providers originating from a wide array of cultural, linguistic, and educational backgrounds. While this diversity enriches clinical practice, it can also pose challenges for patient education delivery [18,19,20]. Differences in communication styles, teaching approaches, and underlying beliefs about health and illness may affect the consistency and effectiveness of PE efforts [21,22]. Moreover, patients may come from backgrounds that differ substantially from those of their providers, widening the gap in understanding, trust, and responsiveness to educational interventions [20,23].

These contextual realities heighten the importance of examining how PE is practiced within Saudi Arabia and similar multicultural healthcare systems. Similar challenges have been reported in neighboring countries. For example, Hayek et al. (2025) found that nurses in Palestinian hospitals faced systemic barriers—including time constraints, staff shortages, and unsuitable environments—while identifying facilitators such as educational technologies and institutional support [24]. Their findings reinforce the regional relevance of addressing organizational and cultural determinants of effective patient education. There is a need to better understand how barriers and facilitators to PE operate in such diverse settings and to identify strategies that support culturally competent, equitable, and effective education. In doing so, healthcare leaders can develop targeted interventions, policies, and professional development initiatives that strengthen the quality of PE, promote adherence, and improve outcomes—particularly for patients managing chronic diseases.

## 2. Aim of the Study

This study aimed to identify barriers and facilitators influencing patient education among nurses in Saudi Arabia hospitals, to inform strategies for improving PE in multicultural settings.

## 3. Methods

### 3.1. Study Design

This study employed a descriptive cross-sectional design to examine the barriers and facilitators influencing the practice of patient education among nurses. This design was chosen to capture a snapshot of nurses’ perceptions across multiple hospital settings at a single point in time, making it suitable for identifying prevalent attitudes and institutional dynamics in real-world healthcare environments.

### 3.2. Setting and Population

The study was conducted across several government and private hospitals in Saudi Arabia that provide a wide range of healthcare services, including tertiary, secondary, and general care. These hospitals serve a diverse patient population and employ a multicultural nursing workforce, reflecting the broader healthcare landscape in the Kingdom.

Eligible participants were registered nurses with at least six months of clinical experience at their current facility and actively engaged in direct patient care. Nurses with less than six months of experience were excluded to ensure that respondents had sufficient familiarity with institutional routines and patient education practices. Recruitment was facilitated through nursing administration offices in each hospital, which distributed information about the study and encouraged voluntary participation.

### 3.3. Sample Size and Sampling Technique

The sample was obtained using a convenience sampling approach. All nurses who met the inclusion criteria were invited to participate. A total of 350 questionnaires were distributed across the participating hospitals, and 289 were returned fully completed, yielding a response rate of 82.6%. Printed copies of the questionnaire were delivered to nursing departments, and nurses were encouraged to complete them during their available time. Multiple reminders were communicated through unit managers and department heads over the two-week data collection period. The final sample of 289 participants was considered sufficient to meet the study objectives and provide representative insights into patient education practices. Although no formal power analysis was conducted, the achieved sample of 289 nurses is comparable to or larger than those used in similar descriptive studies on patient education [15,24]. This sample size was considered adequate to ensure stable estimates and meaningful representation of nurses’ perceptions across different hospital settings.

### 3.4. Data Collection Tools and Procedures

Data were collected using a structured, self-administered questionnaire developed by the research team following a comprehensive review of the literature on barriers and facilitators of patient education. The initial version of the questionnaire was reviewed by a panel of five academic and clinical experts to confirm face and content validity. It was then refined through a focus group discussion with eight clinical nurses drawn from different hospital units to ensure contextual relevance and clarity. Based on their feedback, several items were reworded for clarity, redundant items were removed, and examples were added to enhance comprehensibility. The classification of barriers and facilitators (patient-, nurse-, and organization-related) was informed by previous literature and refined through expert review and the focus group process.

The final questionnaire consisted of three main sections:

1. Demographic Information: age, gender, nationality, marital status, education level, clinical unit, job title, years of experience, and nurse-to-patient ratio.
2. Barriers to Patient Education: 13 items assessing nurse-, patient-, and organization-related barriers.
3. Facilitators of Patient Education: 9 items addressing institutional and professional supports for PE.

All items were rated on a 5-point Likert scale ranging from 1 (strongly disagree) to 5 (strongly agree). Internal consistency was tested in a pilot study of 20 nurses, resulting in a Cronbach’s alpha of 0.86, indicating strong reliability. On average, the questionnaire required 10–15 min to complete.

Data collection was carried out during the last quarter of 2024. The research team visited participating hospitals, distributed printed questionnaires during morning shifts via nurse managers, and later collected the completed forms. Participation was voluntary, with informed consent implied through questionnaire return. Confidentiality and anonymity were maintained throughout the process.

## 4. Data Analysis

Collected data were analyzed using SPSS version 25. Descriptive statistics were used to summarize demographic data and responses to the barrier and facilitator items. Frequencies and percentages were reported for categorical variables, while means and standard deviations were calculated for continuous and Likert-scale data.

For items measured on the 5-point Likert scale, responses were summarized by combining the proportions of participants who selected “Agree” or “Strongly Agree.”

The primary aim of this study was exploratory—focused on describing the overall state of patient education practices and identifying key barriers and facilitators, rather than classifying groups such as gender or nationality in terms of patient education performance. Therefore, no inferential analyses were conducted.

### Ethical Considerations

Ethical approval was obtained from the Research Office—King Abdullah International Medical Research Center (KAIMRC) (Approval Code: roj-data/om/2023/ro/163; Approval Date: 6 November 2023). Administrative permission was also secured from nursing leadership in each participating hospital.

Before participation, nurses were provided with an explanation of the study aims, procedures, and their rights as participants. They were assured that participation was voluntary and that they could withdraw at any time without consequences. Informed consent was obtained through the return of completed questionnaires, which indicated their willingness to participate. Confidentiality and anonymity were strictly maintained, with no identifying information collected. All data were stored securely and were accessible only to the research team.

## 5. Results

### 5.1. Participants’ Demographic Characteristics

A total of 289 nurses participated in the study. The majority of participants were female (92.0%), with only 8.0% being male. In terms of age, the largest group was between 31–40 years (44.6%), followed by those under 30 years (30.1%). Most nurses held a bachelor’s degree (71.3%), while 27.0% had a diploma and only 1.7% held a master’s degree.

Participants worked across various clinical units, with the highest representation from outpatient departments (16.6%), followed by CCU (10.7%) and medical wards (12.5%). Most participants were either single (48.1%) or married (47.4%). A significant majority were non-Saudi nationals (76.1%).

Regarding clinical positions, 95.9% were Staff Nurses, while a small percentage held coordinator or managerial roles. Most nurses had between 1 to 10 years of experience (54.3%), and 76.5% reported working under a 1:5 nurse-to-patient ratio, while 23.5% reported working in higher ratios. Table 1 presents the demographic data of study participants.

### 5.2. Barriers to Patient Education

Participants identified multiple barriers affecting their ability to educate patients, which were grouped into three categories: patient-related, nurse-related, and organizational barriers.

Among patient-related barriers, the most frequently reported was the language difference between nurses and patients, cited by 64.3% of respondents. Cultural differences were also significant (59.2%), followed by patients’ lack of motivation to learn (41.6%). Other notable barriers included patients’ mental or physical limitations (37.3%) and a lack of trust in nurses (26.0%).

Regarding nurse-related barriers, the most prevalent issues were a shortage of nursing staff (72.4%) and heavy workloads (72.0%). Time constraints were also a major concern, identified by 59.9% of nurses. A smaller proportion noted a lack of sufficient awareness, confidence, or knowledge (33.2%) and the perception that patient education is not a priority (13.8%).

Organizational barriers were also substantial. Approximately 39.4% of nurses indicated insufficient availability of educational resources, 37.3% reported frequent staff rotations between wards, and 35.6% cited an unsupportive work environment. Table 2 presents the barriers to PE from the nurses’ perspectives.

### 5.3. Facilitators to Patient Education

Nurses identified several key facilitators that support effective patient education practices. The most commonly reported facilitator was the availability of policy and procedures related to patient education, noted by 63.6% of participants. Similarly, training programs aimed at enhancing patient education skills were cited by 63.7% of nurses.

Other important facilitators included planning education into the workflow (61.9%), availability of clinical nurse resources in each ward (60.6%), and sufficient time allocation for patient education (57.1%). Nurses also highlighted the value of written educational materials (57.8%) and educational resources (58.5%) in facilitating their teaching efforts.

Additionally, 53.0% of nurses indicated that including patient education in nurse appraisal systems and the availability of motivational awards could further incentivize and improve education delivery. Table 3 presents the facilitators to PE from the nurses’ perspectives.

## 6. Discussion

This study explored the barriers and facilitators to patient education (PE) among nurses in Saudi hospitals. The findings reveal that although nurses strongly recognize the importance of PE, several patient-related, nurse-related, and organizational factors significantly impede its delivery. Conversely, the study also identified key institutional and professional facilitators that could enhance PE implementation if adequately supported.

Applying the Donabedian Model of Quality of Care provides a coherent framework for interpreting these findings across the dimensions of structure, process, and outcome. Structural factors refer to the institutional and organizational conditions shaping patient education—such as staffing levels, workload, language diversity, and availability of resources. Process factors encompass the actual behaviors and practices of nurses, including communication, cultural sensitivity, and the integration of education into daily workflow. Outcomes, in this context, relate to the effectiveness of patient education as reflected in improved understanding, adherence, and patient engagement.

### 6.1. Patient-Related Barriers

Language differences and cultural mismatch were the most frequently cited barriers, with nearly two-thirds of nurses identifying them as major obstacles. These findings point to structural challenges within the healthcare system that affect the quality and accessibility of communication. The multicultural composition of the Saudi healthcare workforce and patient population underscores the need for translation services, bilingual staff, and culturally adapted materials to promote mutual understanding and trust [13,25].

### 6.2. Nurse-Related Barriers

Nurses reported time constraints, workforce shortages, and heavy workloads as major process-related barriers to effective patient education. Such conditions disrupt the teaching process and limit opportunities for meaningful patient interaction. The absence of systematic expectations—such as including patient education in performance appraisals—further weakens its perceived importance [15,26]. Consistent with these results, Hayek et al. (2025) also found that time limitations and high patient loads were major obstacles among Palestinian nurses, suggesting that similar workforce structures may affect the quality and consistency of PE delivery across the region [24].

### 6.3. Organizational Barriers

Organizational factors such as unsupportive environments, insufficient educational resources, and frequent staff rotations represent weaknesses in both structure and process. These issues disrupt continuity of care and hinder the development of consistent educational routines. Addressing such challenges requires system-level reforms, including adequate staffing, protected time for education, and improved access to standardized materials. Similar findings from international studies confirm that supportive infrastructure, leadership commitment, and integrated policies are essential to embedding education within routine nursing practice [17,27].

### 6.4. Facilitators to Patient Education

Encouragingly, several facilitators were strongly endorsed by nurses, including the availability of policy and procedure documents, provision of training, and access to written educational materials. These align with international findings that emphasize the value of standardized protocols, ongoing professional development, and accessible tools in enhancing patient education delivery [14].

Moreover, institutional recognition—such as incorporating PE into nurse evaluations and providing motivational incentives—was viewed as an important yet underutilized strategy. These findings support previous recommendations for embedding PE into core nursing competencies and appraisal systems to elevate its status in clinical settings [28,29].

### 6.5. Implications for Nursing Practice and Management in Saudi Arabia

The findings highlight the need for a more integrated and system-oriented approach to strengthen patient education within Saudi hospitals. Incorporating patient education into nursing policies, job descriptions, and performance evaluations would formally establish it as a structural element of care quality. Workforce development should include targeted training in communication, cultural competence, and digital education tools. Given that nearly one-third of nurses reported lacking sufficient awareness, confidence, or knowledge, and about 14% did not consider patient education a priority, nursing education institutions should also strengthen curricula to better prepare nurses for this role. Additionally, improving staffing levels and providing protected time for education are critical structural investments that enhance care quality. Leadership support, multilingual resources, and culturally tailored education programs can further improve the process and outcomes of patient education.

### 6.6. Theoretical Framing and Broader Significance

The Donabedian Model offers a strong foundation for understanding how the identified barriers and facilitators interact to influence patient education outcomes. The study demonstrates that improving structure—through policies, staffing, and resources—directly enhances the process of education delivery, ultimately leading to better outcomes such as patient understanding, adherence, and empowerment. Strengthening any one dimension reinforces the others, underscoring the importance of system-level alignment for effective patient education.

While this study was conducted in Saudi hospitals, its implications extend cautiously beyond the national context. The Gulf and broader Middle Eastern healthcare systems share many demographic and workforce characteristics, including multicultural nursing staff, diverse patient populations, and similar institutional structures. For this reason, the findings may be informative for comparable regional contexts; however, their generalizability remains limited and should be interpreted with caution given the use of convenience sampling.

Future research should build on these findings by incorporating patient perspectives, conducting interventional studies to test targeted strategies, and evaluating the impact of policy changes at the institutional and system levels. Integrating patient education more explicitly into undergraduate nursing curricula may also help prepare the future workforce to address persistent barriers such as trust, cultural differences, and communication challenges.

## 7. Limitations

This study has several limitations that should be considered when interpreting its findings. The cross-sectional design captures nurses’ perceptions at a single point in time and does not allow for causal inferences or insights into changes over time. Additionally, the use of self-administered questionnaires introduces the potential for response bias, including recall and social desirability bias, which may have influenced participants’ responses. While the sample size was statistically adequate and representative of the study setting, a larger and more diverse sample could have provided greater certainty and enhanced the robustness of subgroup comparisons. The sample was predominantly female (92%); however, this distribution reflects the actual gender profile of the nursing workforce in Saudi Arabia and internationally, where nursing remains a female-dominated profession. In addition, the cultural and linguistic diversity of the workforce may have influenced how nurses perceived and reported barriers and facilitators to patient education, introducing potential bias into the findings. Furthermore, the study focused exclusively on nurses’ perspectives without incorporating patient viewpoints, which may have limited the breadth of understanding of the patient education process. Lastly, this study relied solely on descriptive analysis, as it was exploratory in nature and aimed to describe the overall state of patient education rather than classifying groups based on sociodemographic variables; future studies should incorporate inferential analyses to explore these associations.

## 8. Conclusions

This study provides important insights into the multifaceted barriers and facilitators influencing patient education among nurses in hospitals in Saudi Arabia. Despite strong professional acknowledgment of the value of patient education, its implementation is frequently compromised by time constraints, staffing shortages, organizational limitations, and communication challenges—particularly in a culturally and linguistically diverse workforce. On the other hand, the availability of policies, training, and structured educational resources is recognized as a key enabler of effective patient teaching.

The findings underscore the need for healthcare institutions in Saudi Arabia and similar contexts to adopt comprehensive, system-level strategies that integrate patient education into nursing roles, performance appraisals, and workflow planning. Addressing these barriers and strengthening identified facilitators is essential for improving patient engagement, treatment adherence, and long-term health outcomes. Ultimately, enhancing the delivery of patient education represents a vital step toward achieving high-quality, culturally competent, and patient-centered care.

## Figures and Tables

**Table 1:** Demographic data of study participants.

Variable	Category	N (%)
Age Group	Less than 30 yrs	87 (30.1%)
	31–40 yrs	129 (44.6%)
	41–50 yrs	50 (17.3%)
	51–60 yrs	21 (7.3%)
	Above 60 yrs	2 (0.7%)
Gender	Female	266 (92.0%)
	Male	23 (8.0%)
Education Level	Diploma	78 (27.0%)
	Bachelor	206 (71.3%)
	Master	5 (1.7%)
Unit/Ward	Pediatric Wards	55 (19.0%)
	Maternity Wards	54 (18.7%)
	Medical and Surgical Wards	144 (49.8%)
	Other (i.e., OPD, ICUs, etc.)	36 (12.5%)
Marital Status	Single	139 (48.1%)
	Married	137 (47.4%)
	Divorced	10 (3.5%)
	Widowed	3 (1.0%)
Nationality	Saudi	69 (23.9%)
	Non-Saudi *	220 (76.1%)
Nursing Position	Clinical Nurse Coordinator	8 (2.8%)
	Staff Nurse	277 (95.9%)
	Nurse Manager	4 (1.4%)
Years of Experience	1–5 yrs	78 (27.0%)
	6–10 yrs	79 (27.3%)
	11–15 yrs	60 (20.8%)
	16–20 yrs	32 (11.1%)
	Above 20 yrs	40 (13.8%)

* Non-Saudi nurses included mainly Filipino, Indian, Jordanian, and Egyptian nationals, reflecting the multicultural composition of the Saudi nursing workforce.

**Table 2:** Barriers to PE from the nurses’ perspectives.

Domain	Barrier	Agreement N (%)
Patient	Patient does not speak the same language	186 (64.3%)
	Patient has a different cultural background	171 (59.2%)
	Patient lacks motivation to learn	120 (41.6%)
	Patient has mental or physical limitations	108 (37.3%)
	Patient does not trust nurses	75 (26.0%)
Nurse	Nurse shortage	209 (72.4%)
	Heavy workload	208 (72.0%)
	Time constraints	173 (59.9%)
	Lack of awareness, confidence, or knowledge	96 (33.2%)
	Patient education is not a priority	40 (13.8%)
Organization	Insufficient educational resources	114 (39.4%)
	Frequent rotation of nurses between wards	108 (37.3%)
	Unsupportive environment	103 (35.6%)

**Table 3:** Facilitators to PE from the nurses’ perspectives.

Facilitator	Agreement N (%)
Offering training programs for patient education	184 (63.7%)
Availability of policy and procedures for patient education	184 (63.6%)
Planning patient education into workflow	179 (61.9%)
Availability of clinical nurse resources in each ward	175 (60.6%)
Availability of educational resources	169 (58.5%)
Providing written educational materials	167 (57.8%)
Providing the nurse with enough time	165 (57.1%)
Considering patient education in nurse appraisal	153 (53.0%)
Availability of motivational awards	153 (53.0%)

## Data Availability

The data presented in this study are available on reasonable request from the corresponding author. The data are not publicly available due to privacy and ethical restrictions.

## Public Involvement Statement

No public involvement in any aspect of this research.

## Guidelines and Standards Statement

This manuscript was drafted against the Strengthening the Reporting of Observational Studies in Epidemiology (STROBE) statement for cross-sectional research [30].

## Abbreviations

The following abbreviations are used in this manuscript:

PE	Patient Education
